# Revealing and engineering contact-origin noise in ultrathin tellurium transistors

**DOI:** 10.1039/d5na01062d

**Published:** 2026-03-13

**Authors:** Hae-Won Lee, Minjae Kim, Junho Ban, Jae Hyeon Jun, Kiyung Kim, Useok Choi, Jung Tae Lee, Byoung Hun Lee

**Affiliations:** a Department of Electrical Engineering, Pohang University of Science and Technology Cheongam-ro 77, Nam-gu Pohang Gyeongbuk 37673 Republic of Korea bhlee1@postech.ac.kr; b National Institute for Nanomaterials Technology, Pohang University of Science and Technology Cheongam-ro 77 Nam-gu Pohang Gyeongbuk 37673 Republic of Korea; c Graduate School of Semiconductor Technology, Pohang University of Science and Technology Cheongam-ro 77 Nam-gu Pohang Gyeongbuk 37673 Republic of Korea

## Abstract

Tellurium (Te) has emerged as a promising p-type semiconductor for ultrathin electronics owing to its strong air stability, excellent hole transport, narrow bandgap, and BEOL-integration compatibility. However, when the Te thickness approaches the depletion width, traps at the contact interface strongly affect carrier injection and introduce excess low-frequency noise. Here, we systematically investigate the origin of noise in ultrathin Te field-effect transistors (FETs) through bias- and temperature-dependent 1/f noise analysis. In devices with a 5 nm Te channel, contact-origin trap-assisted tunneling dominates in the low-current regime, producing deviations from the carrier-number-fluctuation (CNF) model at 300 K. Cooling to 100 K suppresses trap activation and restores typical CNF behavior, whereas 13 nm devices maintain CNF consistency at both temperatures due to screening of the contact region. To mitigate contact-origin noise, a locally thickened (13 nm) Te layer was inserted beneath the source and drain metal contact while preserving a 5 nm active Te channel. This design restores CNF behavior at room temperature, lowers the noise level in the nA current regime by an order of magnitude, and decreases the drain-bias dependence of noise by approximately twofold. The results identify near-contact traps as the primary noise source in ultrathin Te and demonstrate contact-centric engineering as an effective strategy to decouple device scaling from noise, enabling reliable, low-noise Te electronics.

## Abstract 2

This study identifies the origin of excess low-current noise in ultrathin Te FETs and suppresses it by locally modulating the Te thickness near the contacts.

## Graphical abstract

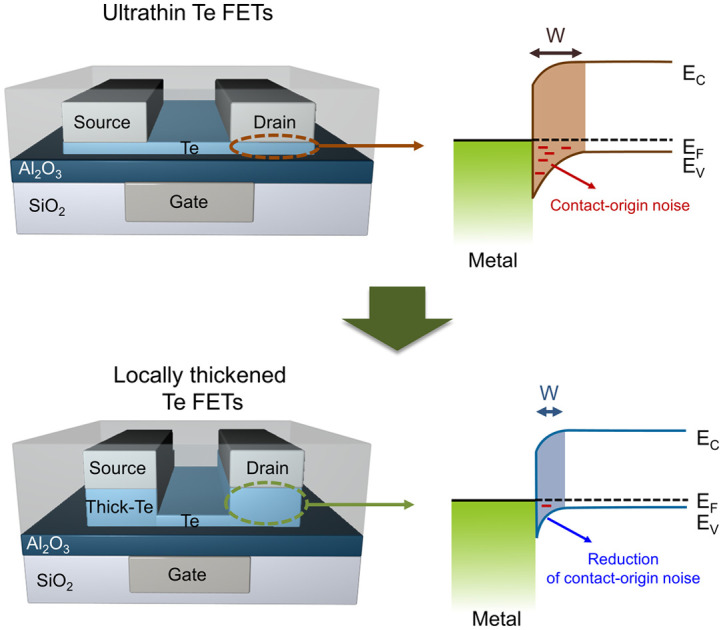

## Introduction

Tellurium (Te) has emerged as a promising p-type semiconductor for ultrathin electronics. It can be integrated in BEOL-compatible flows with a low thermal budget, maintains efficient hole transport down to a channel thickness of a few nanometers, and exhibits strong broadband absorption owing to its narrow bandgap.^1–4^ When integrated with n-type oxides such as IGZO or ZnO, Te enables complementary metal-oxide-semiconductor (CMOS) architectures suited for large-area and heterogeneous integration.^5–7^ Reducing the channel thickness to the few-nanometer regime enhances electrostatic control and subthreshold swing, lowers switching charge and dynamic power, and facilitates easy integration with interconnects and sensors.^8–12^ For photodetectors and mixed-signal front ends, ultrathin channels further promote strong gate coupling and high responsivity under modest bias.^13–15^

As the channel thickness approaches the depletion width, contact–channel coupling becomes significant, and defects at the metal/Te interface increasingly govern carrier transport through trap-assisted mechanisms.^16–18^ These interface traps modulate the Schottky barrier height and the carrier injection rate, exhibiting strong dependence on gate voltage (*V*_G_), drain voltage (*V*_D_), and temperature.^19,20^ Although contact engineering for defect reduction, such as ultrathin interlayer insertion, contact doping has been widely explored, the impacts on noise characteristics have not been systematically investigated.^21,22^ Since defect-induced performance fluctuations not only degrade device-level parameters but also translate into circuit-level limitations, manifesting as reduced signal-to-noise ratio (SNR) and effective number of bits (ENOB) in analog and mixed-signal systems, it is imperative to have a holistic approach that includes the study of the noise characteristics.^23–26^ Recently, 1/f noise in Te-based p-type field-effect transistors (FETs) has been studied, but those studies focused on thick channel regime above 10 nm—leaving the ultrathin (∼5 nm) regime underexplored.^27–29^ In this context, Te exhibits a thickness dependence in its electronic properties, such that in the ultrathin thickness, the injection region becomes increasingly sensitive to the near-contact environment and metal/Te interfacial states—making Te a suitable platform for investigating and mitigating contact-origin noise mechanisms.

In this work, we perform bias- and temperature-dependent 1/f noise measurements to investigate carrier transport at the metal/Te interface. Te FETs with 5 nm and 13 nm channels were characterized at 300 K and 100 K. At 300 K, the thinner 5 nm devices exhibit increased noise and pronounced *V*_D_ dependence, particularly in the low-bias regime, deviating from the carrier-number-fluctuation (CNF) model—evidence of strong trap influence. In the nA regime, the measured spectral density is ∼15 times higher than the value predicted by the CNF model; the gap decreases with increasing current, and the spectral density aligns with CNF for currents above 10^−7^ A. These effects disappear at 100 K and are absent in 13 nm devices at both temperatures. Based on these observations, we propose selectively increasing the Te thickness beneath the source and drain contacts to suppress contact-origin noise and reduce contact resistance. With this strategy, the noise level in the nA current range is reduced by a factor of 10 compared with 5 nm Te. This approach highlights the importance of contact engineering in advancing ultrathin semiconductors toward high-performance, low-noise nanoelectronics.

## Experimental section

### Device fabrication and structural characterization

p-type Te FETs were fabricated with a buried-gate architecture comprising a bilayer metal stack of Ti (10 nm) as an adhesion layer and Ni (60 nm) deposited by e-beam evaporation. A 12 nm Al_2_O_3_ gate dielectric was grown on the gate metal by atomic layer deposition (ALD) using trimethylaluminum (TMA) and H_2_O at 200 °C. Subsequently, 5 nm and 13 nm Te layers were deposited on the Al_2_O_3_ by RF sputtering from a high-purity Te target (99.999%, iTASCO) and patterned by reactive-ion etching (RIE) in an Ar/CF_4_ gas mixture. The Te films were then annealed at 150 °C for 1 hour in ambient air to promote crystallization. Ni source/drain contacts were formed by e-beam evaporation and defined *via* lift-off. The thick-Te contacts were implemented as follows: after source/drain patterning for lift-off, an additional 13 nm Te layer was sputtered only in the contact regions, followed by Ni deposition and lift-off definition. Finally, the devices were passivated by depositing a 10 nm Al_2_O_3_ capping layer *via* ALD at 150 °C. The morphological features and precise thicknesses of the Te channels were characterized using atomic force microscopy (AFM; Jupiter XR, Oxford Instruments). As shown in Fig. S1 (SI), the measured thicknesses for both the 5 nm and 13 nm films were consistent with our design parameters.

### Electrical measurements

Electrical characteristics, including transfer curves, were measured using a semiconductor parameter analyzer (Keithley 4200-SCS). For the 1/f noise characterization, the gate bias was provided by the Keithley 4200-SCS, while the drain terminal was biased and the resulting current was amplified using a low-noise current preamplifier (SR570, Stanford Research Systems) operated in low-noise mode. The amplified drain-current fluctuations were converted into a voltage signal and subsequently processed by a dynamic signal analyzer (35670A, Agilent) to obtain the drain-current noise power spectral density (*S*_ID_) *via* fast Fourier transform (FFT) analysis. To suppress external interference and ground-loop noise, the entire measurement system was configured with a single-point common ground. The noise spectra were acquired over a frequency range of 5 Hz to 1.605 kHz, with each spectrum being the result of 10 averaged FFT acquisitions to ensure statistical significance. Unless otherwise specified, the noise magnitudes reported in this work were evaluated at a representative frequency of 10 Hz. For low-temperature characterization, the measurement chamber was cooled with liquid nitrogen, and the temperature was precisely controlled using a temperature controller (MSTECH, MST-1000H).

## Results and discussion

**Fig. 1:**
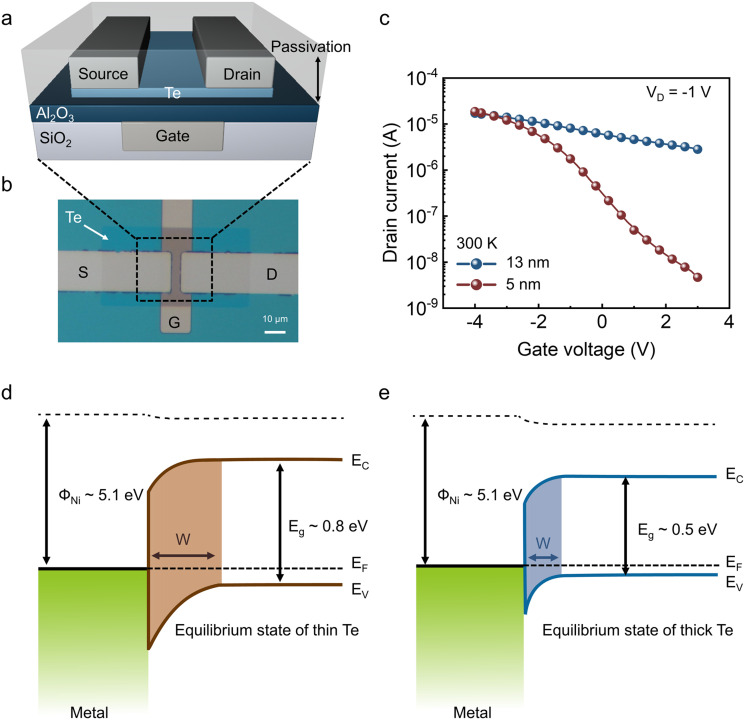
(a) Schematic of the fabricated p-type Te FETs. (b) Optical microscope image of a device with channel length (*L*) = 5 µm and width (*W*) = 20 µm. (c) Transfer characteristics of 5 nm and 13 nm Te FETs measured at *V*_D_ = −1 V and 300 K. Schematics of the equilibrium band alignment for (d) 5 nm and (e) 13 nm Te contacted by a metal.

Fig. 1a shows the schematic of the fabricated p-type Te FETs. To ensure a uniform electric field across the channel, Ti (10 nm)/Ni (60 nm) gate electrodes were embedded in a SiO_2_ trench.^31^ A 12 nm Al_2_O_3_ layer was deposited on the buried gate structure by ALD as the gate dielectric, followed by sputter deposition of a 5 nm or 13 nm Te film, which was patterned by RIE. E-beam-evaporated Ni source and drain contacts were formed by the lift-off process. Then, the Te channel region was passivated with an Al_2_O_3_ layer to prevent the influence of ambient air.^16^Fig. 1b presents an optical image of the devices, featuring a channel width of 20 µm and a length of 5 µm. Fig. 1c shows the transfer characteristics of Te FETs with channel thicknesses of 5 nm and 13 nm. Due to quantum confinement, the Te bandgap widens as the thickness decreases, with reported values of approximately 0.8 eV for 5 nm and 0.5 eV for 13 nm Te.^30^ The 5 nm device exhibits a field-effect mobility of 3.86 cm^2^ V^−1^ s^−1^ and an on/off ratio of 3.98 × 10^3^. In contrast, the 13 nm device shows a lower mobility of 2.21 cm^2^ V^−1^ s^−1^, a higher off-state current, and an on/off ratio of 6.05 owing to its narrower bandgap.

Fig. 1d and e illustrate the equilibrium band alignments for 5 nm and 13 nm Te contacted by Ni. In two-dimensional semiconductors like Te, reducing the thickness not only widens the bandgap but also lowers the carrier concentration.^32,33^ Consequently, the junction-induced band bending extends over a wider depletion region in the 5 nm film (Fig. 1d), whereas the 13 nm Te, with higher carrier density, shows a narrower depletion region (Fig. 1e). Because Te typically forms Schottky contacts and its electronic properties vary strongly with thickness, contact-related parameters—such as depletion width, Schottky barrier height, and leakage current—also change significantly.

**Fig. 2:**
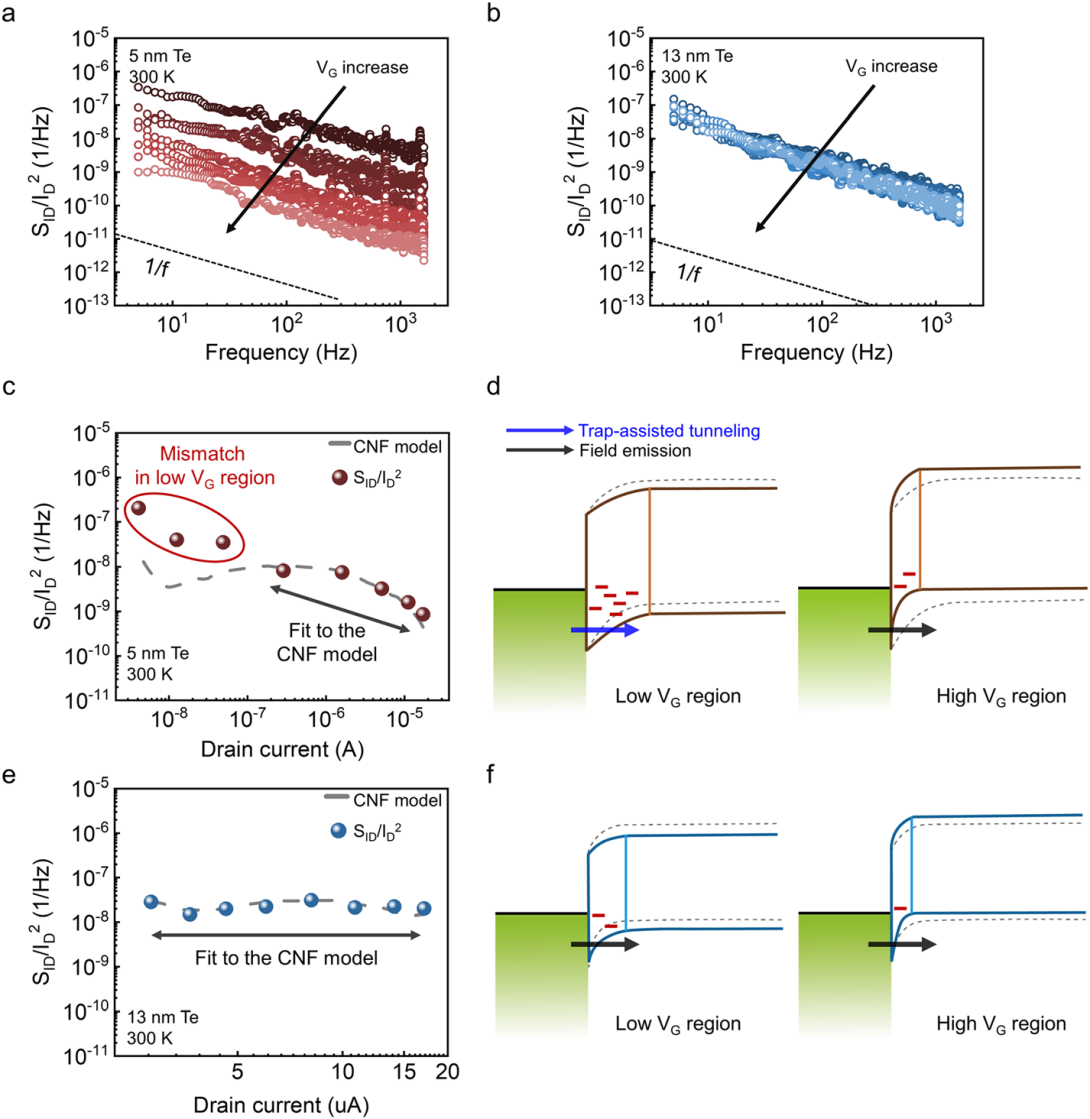
*S*_ID_/*I*_D_^2^ as a function of frequency measured at *V*_D_ = −1 V under gate voltages *V*_G_ = 3 V to *V*_G_ = −4 V in −1 V steps for (a) 5 nm Te FETs and (b) 13 nm Te FETs. *S*_ID_/*I*_D_^2^ at 10 Hz as a function of *I*_D_ for the (c) 5 nm Te FETs and (e) 13 nm Te FETs. Schematic energy–band diagrams of the metal/Te contact in the low- and high-*V*_G_ regions for (d) 5 nm and (f) 13 nm Te FETs.

Fig. 2 presents the 1/f noise characteristics of 5 nm and 13 nm Te FETs measured at 300 K under various gate biases. Fig. 2a and b show the bias dependence of the noise spectra at *V*_D_ = −1 V, with *V*_G_ swept from 3 V to −4 V in −1 V steps. For both devices, the noise spectral density follows an approximate 1/f^α^ dependence (*α* ≈ 1) across the measured frequency range. In the 5 nm Te FETs, the normalized spectral density at 10 Hz decreases by more than two orders of magnitude as *V*_G_ is swept from 3 V to −4 V (Fig. 2a). In contrast, the 13 nm device varies within one order of magnitude over the same range (Fig. 2b). Thus, the 5 nm device shows a much stronger gate dependence and higher noise level in the low-*V*_G_ regime.

We investigated the correlation between drain current and noise level to elucidate the defect-related transport mechanism in Te FETs. Fig. 2c plots the normalized drain-current noise magnitude as a function of drain current for the 5 nm Te FETs measured at 300 K. The dashed lines represent the CNF model, which attributes 1/f noise to charge trapping and de-trapping at the channel/dielectric interfaces.^34–36^ The normalized drain-current noise spectral density based on the CNF model^25^ is expressed as:1
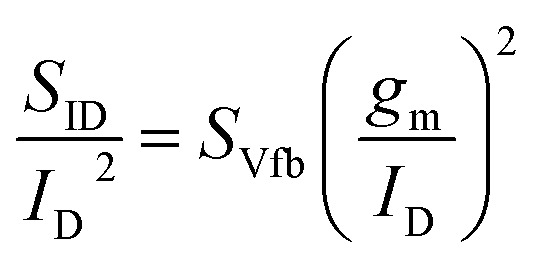
where *S*_Vfb_ represents the flat-band voltage fluctuation spectral density. To evaluate the noise mechanism, we generated CNF reference curves (gray lines) by plotting *A*_CNF_(*g*_m_/*I*_D_)^2^. Here, *g*_m_ was extracted directly from the measured *I*_D_–*V*_G_ characteristics, and *S*_ID_/*I*_D_^2^ was evaluated at 10 Hz. The plotting prefactor *A*_CNF_ was determined by matching the model to the experimental data in the CNF-dominant regime. This benchmarking approach allows for a clear distinction between CNF-governed transport and deviations caused by other noise components without invoking additional fitting parameters. The normalized current spectral density at 10 Hz agrees well with the CNF model in the high-current regime (>10^−7^ A), indicating CNF-dominated behavior. However, at lower drain currents (<10^−7^ A), the measured noise deviates significantly from the CNF prediction—by approximately 15-fold in the nA regime—revealing the presence of additional noise components. Fig. 2d illustrates the band diagrams as a function of *V*_G_, where the dashed line denotes the equilibrium condition. Defect states associated with TeO_*x*_ are present near the Te/metal contact region, influencing carrier injection. As a result, even in the presence of Schottky contacts, trap-assisted tunneling (TAT) through these defects can dominate transport and contribute to the elevated off-current.^19^ At low *V*_G_, the depletion region widens, exposing carriers to a greater density of active traps that modulate the Schottky barrier; their capture–emission dynamics induce current fluctuations. Consequently, TAT is enhanced and the measured noise exceeds the level predicted by the CNF model. At high *V*_G_, the depletion width contracts, reducing carrier–trap interactions and suppressing TAT-related noise. Under these conditions, field emission becomes the dominant conduction mechanism, and no additional noise components beyond the CNF model are observed. For the 13 nm Te FETs, the noise behavior follows the CNF model across the entire *V*_G_ range (Fig. 2e), indicating that channel-origin fluctuations prevail over contact-related effects. The narrower depletion region reduces the number of active contact-side traps at both low and high *V*_G_, keeping conduction dominated by field emission. As a result, contact-origin noise is suppressed, and the measured spectral density remains consistent with the CNF model (Fig. 2f).

**Fig. 3:**
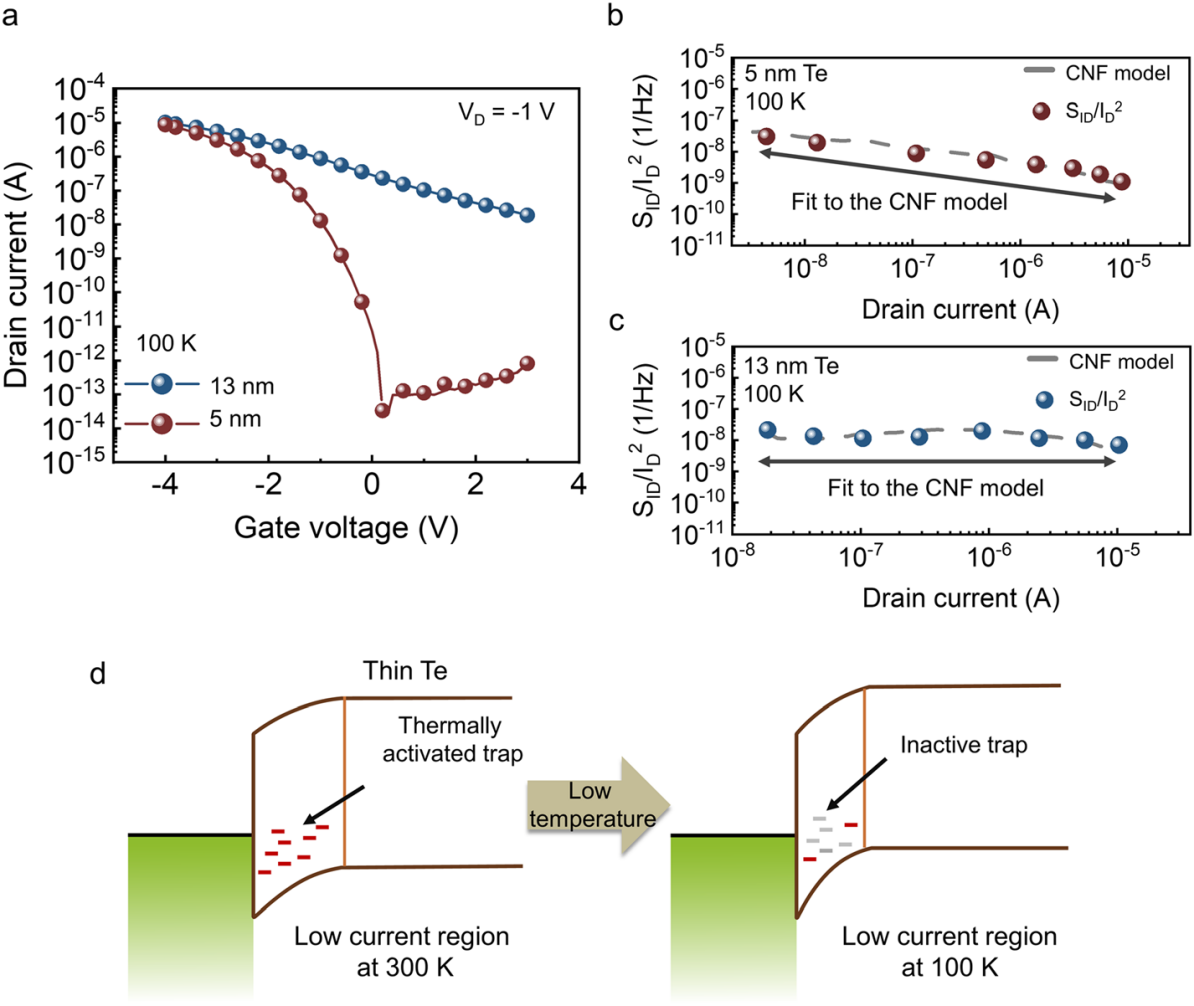
(a) Transfer characteristics of 5 nm and 13 nm Te FETs measured at *V*_D_ = −1 V and 100 K. *S*_ID_/*I*_D_^2^ at 10 Hz as a function of *I*_D_ for the (b) 5 nm Te FETs and (c) 13 nm Te FETs at 100 K. (d) Schematic energy–band diagrams of the metal/Te contact in the low current regions for 5 nm Te FETs at 300 K and 100 K.

The temperature dependence of the traps responsible for the additional low-current noise in the 5 nm Te FETs is further investigated. Fig. 3a presents the transfer characteristics of 5 nm and 13 nm Te FETs measured at 100 K. The 5 nm device exhibits a field-effect mobility of 3.9 cm^2^ V^−1^ s^−1^ and an on/off ratio of 3.4 × 10^8^, approximately five orders of magnitude higher than at 300 K. The 13 nm device shows a mobility of 2.93 cm^2^ V^−1^ s^−1^ and an on/off ratio of 5.47 × 10^2^, representing an improvement of about two orders of magnitude over its 300 K value. In both devices, the off-current decreases at low temperature due to suppressed thermally activated carriers and reduced thermally assisted leakage pathways.

Fig. 3b and c plot the normalized drain-current noise as a function of drain current at 100 K. Unlike at 300 K, where the 5 nm device exhibited excess noise beyond the CNF model at low current, the 5 nm Te FETs now follow the CNF prediction across the entire current range (Fig. 3b). The 13 nm Te FETs adhere to the CNF model at both 300 K and 100 K over all current levels (Fig. 3c), consistent with its narrower depletion region and correspondingly weaker interaction with contact-side traps.

At 300 K, in the low *V*_G_ regime of the 5 nm Te FETs, carrier injection at the contact is dominated by TAT enabled by thermally activated traps. At 100 K, these traps become thermally inactive, suppressing TAT and making the Schottky barrier the limiting factor for carrier injection, which lowers the off-current. The difference in additional low-current noise (<10^−7^ A) between 300 K and 100 K therefore originates from the thermal activation of contact-interface traps. At 100 K, the low *V*_G_ (<10^−7^ A) condition narrows the depletion region, reducing the effective trap population compared with 300 K. However, since the device operates below *V*_TH_ with a weak gate field, the depletion width does not shrink sufficiently to remove carrier–trap interactions entirely. Consequently, although the depletion region remains relatively wide, the traps are no longer thermally active, and their participation in transport is diminished, resulting in the absence of additional trap-induced noise in the low-current regime (Fig. 3d).

In Fig. 2 and 3, the excess noise observed in the low-current regime of the 5 nm Te device was attributed to defects at the contact interface. Because the drain field directly modulates the barrier width and influences field emission and TAT, noise originating from contact-interface defects is expected to depend on the lateral electric field. To verify this, we analyzed the noise as a function of *V*_D_. For the 5 nm Te FETs, measurements were conducted at *V*_G_ = 3 V and *V*_G_ = −4 V at 300 K and *V*_G_ = −0.5 V and *V*_G_ = −4 V at 100 K. For the 13 nm Te FETs, measurements were conducted at *V*_G_ = 3 V and *V*_G_ = −4 V at both temperatures. For both devices, *V*_D_ was swept from −0.25 V to −1 V in −0.25 V steps.

**Fig. 4:**
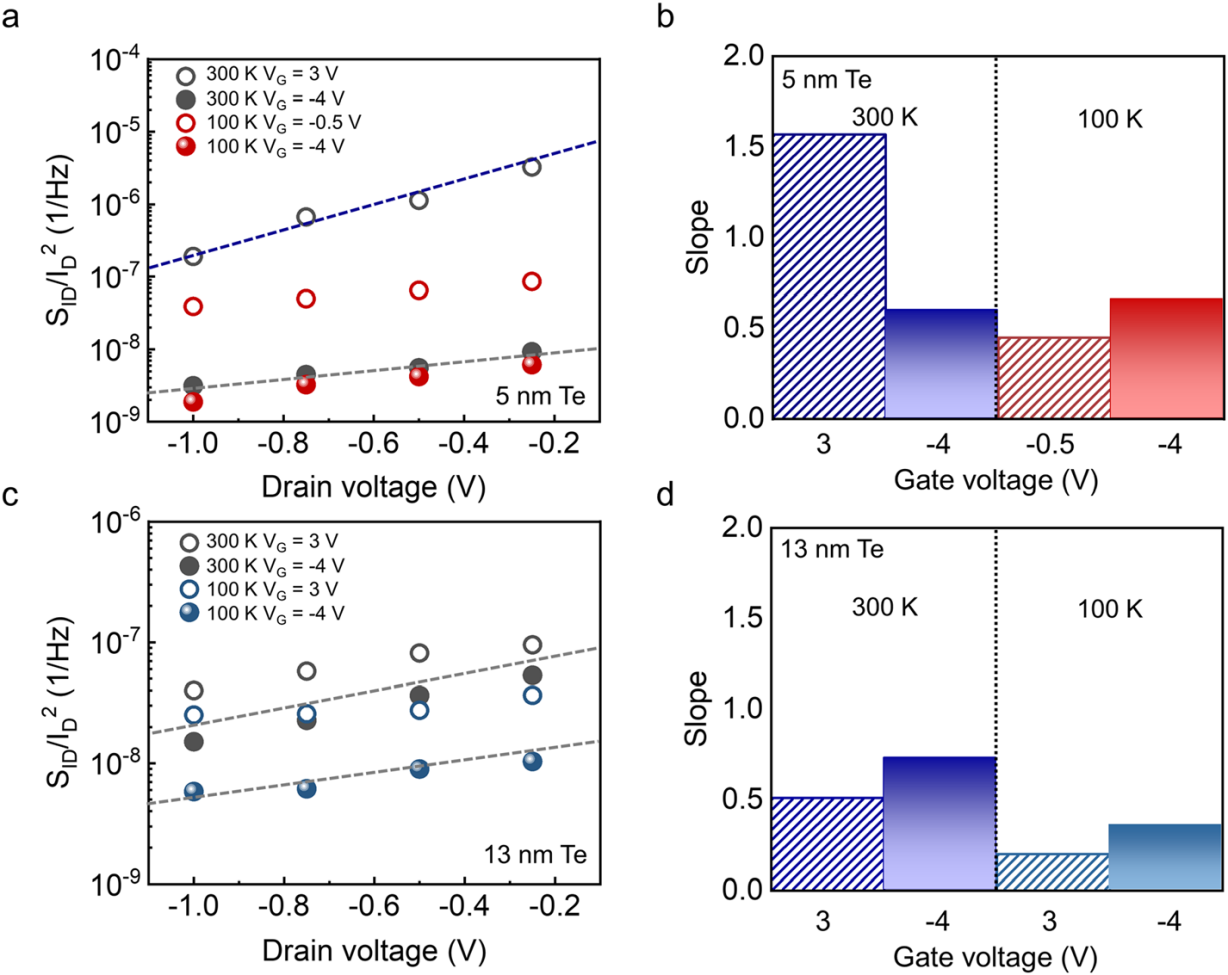
(a) *S*_ID_/*I*_D_^2^ at 10 Hz as a function of *V*_D_ for the 5 nm Te FETs. (b) *V*_D_ dependence slope of *S*_ID_/*I*_D_^2^ at low and high *V*_G_ at 300 K and 100 K for 5 nm Te FETs. (c) *S*_ID_/*I*_D_^2^ at 10 Hz as a function of *V*_D_ for the 13 nm Te FETs. (d) *V*_D_ dependence slope of *S*_ID_/*I*_D_^2^ at low and high *V*_G_ at 300 K and 100 K for 13 nm Te FETs.

Fig. 4a shows the *V*_D_ dependence of the noise level at 10 Hz in the 5 nm Te FETs. At *V*_G_ = 3 V, the noise level rises sharply as ∣*V*_D_∣ decreases, with a slope of ∼1.6. This trend is consistent with a weaker lateral field widening the effective barrier width at the contact, thereby enhancing carrier–defect interactions and elevating the noise at 300 K. At *V*_G_ = −4 V, the barrier width is already thin due to the strong gate field, reducing defect interactions. Consequently, channel-origin noise dominates and the *V*_D_ dependence decreases, yielding a slope of ∼0.6. Consistent with the results in Fig. 3, no additional contact-origin noise appears at 100 K, even in the low-current regime; the slopes at 100 K are ∼0.45 for *V*_G_ = −0.5 V and ∼0.66 for *V*_G_ = −4 V. For the 13 nm Te FETs, the slopes remain below 0.7 at both 100 K and 300 K for *V*_G_ = 3 V and *V*_G_ = −4 V, indicating consistently weak *V*_D_ dependence. Based on the systematic comparison between the 5 nm and 13 nm channels, the dominant noise mechanism is inferred to evolve *via* a gradual thickness-driven crossover. As the Te thickness increases, the relative influence of the near-contact depletion region is mitigated, leading to a progressive reduction in contact-origin noise and a weakened sensitivity to drain bias. This evolution results in a broader regime where the CNF model accurately describes the noise scaling. This transition is not governed by a fixed critical thickness but is determined by the interplay between the channel thickness and the effective depletion width, which depends on the electrostatic environment and interface conditions.

**Fig. 5:**
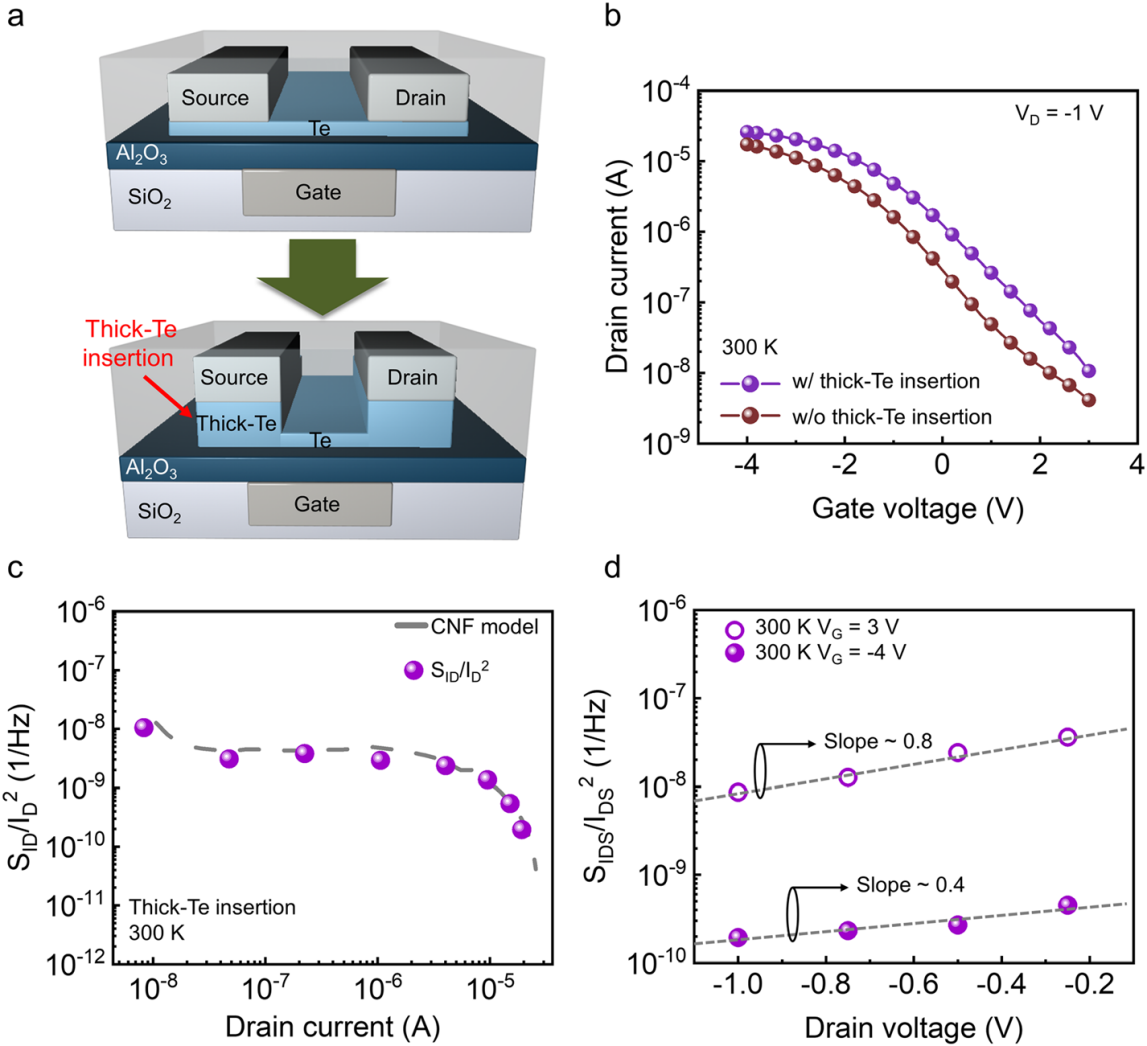
(a) Schematic of the thick-Te insertion at the contact regions to suppress contact-origin noise. (b) Transfer curves for 5 nm Te FETs with and without thick-Te insertion. (c) *S*_ID_/*I*_D_^2^ at 10 Hz as a function of *I*_D_ for the thick-Te inserted FETs. (d) *V*_D_ dependence slope of *S*_ID_/*I*_D_^2^ at 300 K for thick-Te inserted FETs.

From the noise behavior across current regimes as functions of thickness and temperature in Te FETs, we identified additional noise sources that emerge in ultrathin Te channels, governed by the underlying band structure. To suppress the excess noise observed at room temperature in the low-current regime of the 5 nm Te FETs, a thickness-modulated contact geometry was adopted. Specifically, a 13 nm Te layer was selectively introduced under the source and drain contacts, a structure whose experimental feasibility was recently demonstrated through cross-sectional scanning electron microscope (SEM) analysis in our previous work.^30^ This locally thickened contact structure effectively reduces the depletion width at the metal–semiconductor interface, thereby suppressing contact-origin noise (Fig. 5a). Compared with a device using a uniform 5 nm Te channel, the modified Te FETs exhibit a mobility of 4.78 cm^2^ V^−1^ s^−1^ and an on/off ratio of 2.44 × 10^3^ (Fig. 5b). Fig. 5c shows the noise characteristics at 300 K as a function of current level while sweeping *V*_G_ from 3 V to −4 V in −1 V steps. Unlike the all-thin (5 nm) device, the modified structure follows the CNF model even in the low-*V*_G_ region at 300 K. By employing this thickness-modulation strategy, the noise magnitude in the low-current regime (nA range) is reduced by approximately one order of magnitude compared to the uniform 5 nm Te devices. This suppression of noise is consistent with the significantly improved contact characteristics of the locally thickened geometry; as demonstrated in our previous report,^30^ this specific contact structure achieves a low contact resistance (*R*_c_) of 1.5 kΩ µm, which facilitates more efficient carrier injection and minimizes contact-origin fluctuations by mitigating interactions with contact-side defects. Consistently, the slope of the *V*_D_-dependent noise is ∼0.8 at *V*_G_ = 3 V, about half that of the 5 nm device, and ∼0.4 at *V*_G_ = −4 V (Fig. 5d). Consequently, local thickness control at the contact region enabled by selective thickening or interface engineering can effectively decouple channel thickness scaling from noise increase, maintaining an ultrathin active channel while suppressing excess low-frequency noise. These results indicate that the proposed local thickness-engineering approach may be extendable to other ultrathin or 2D semiconductors with electronic characteristics similar to those of Te, particularly a relatively narrow bandgap and contact-limited injection behavior. Nevertheless, because defect formation and metal/semiconductor interfacial interactions are inherently material-specific, the degree of improvement and the optimal implementation are expected to depend on each material's defect landscape and metal/2D interaction pathways.

## Conclusion

In this study, we systematically investigated the low-frequency noise in ultrathin Te FETs. In the 5 nm devices at 300 K, contact-interface traps raise the noise level and impose a strong *V*_D_ dependence in the low-current regime, deviating from the CNF model. By contrast, 13 nm devices retain CNF behavior at both temperatures due to better screening of the contact region. By locally thickening Te under the source/drain contacts, the influence of contact-interface traps could be minimized. Although varying contact metals with different work functions is a useful route to modulate the Schottky barrier height, in ultrathin/2D channels the effective barrier modulation can be limited by Fermi-level pinning and interfacial states. In this context, our thickness-based approach provides an alternative lever by tuning the near-contact depletion condition and contact–channel coupling within the same contact material/process, thereby avoiding confounding changes in interfacial chemistry. Overall, the strong influence of contact-origin trap states as the dominant noise source underscores the importance of optimized contact engineering for achieving high-performance and practical integration of ultrathin Te FETs in future nanoelectronic systems.

## Author contributions

H.-W. L and B. H. L conceived the idea and designed the experiments. B. H. L supports resources. H.-W. L executed experiments and electrical characteristics measurement. H.-W. L and B. H. L cowrote the manuscript, and all authors were involved in the discussions.

## Conflicts of interest

There are no conflicts to declare.

## Supplementary Material

NA-008-D5NA01062D-s001

## Data Availability

All data generated or analyzed during this study are included in this published article. Supplementary information (SI): AFM data confirming the Te film thickness, as shown in Fig. S1. See DOI: https://doi.org/10.1039/d5na01062d.
