# Supplementary material for: Revealing and engineering contact-origin noise in ultrathin tellurium transistors
Source: Nanoscale Adv. 2026 Mar 13;8(8):2566–73. doi: 10.1039/d5na01062d (PMC12984060; doi:10.1039/d5na01062d)
Supplement: NA-008-D5NA01062D-s001 [file NA-008-D5NA01062D-s001.pdf]

Supplementary Information (SI) for Nanoscale Advances.
This journal is © The Royal Society of Chemistry 2026

Supplementary Information

Revealing and Engineering Contact-Origin Noise in Ultrathin Tellurium Transistors

*Hae-Won Lee[a], Minjae Kim[b], Junho Ban[c], Jae Hyeon Jun[b], Kiyung Kim[b], Useok Choi[c], Jung Tae Lee[c] and Byoung Hun Lee[a,b,c]**

[a]Department of Electrical Engineering, Pohang University of Science and Technology, Cheongam-ro 77, Nam-gu, Pohang, Gyeongbuk 37673, Republic of Korea

[b]National Institute for Nanomaterials Technology, Pohang University of Science and Technology, Cheongam-ro 77, Nam-gu, Pohang, Gyeongbuk 37673, Republic of Korea

[c]Graduate School of Semiconductor Technology, Pohang University of Science and Technology, Cheongam-ro 77, Nam-gu, Pohang, Gyeongbuk 37673, Republic of Korea

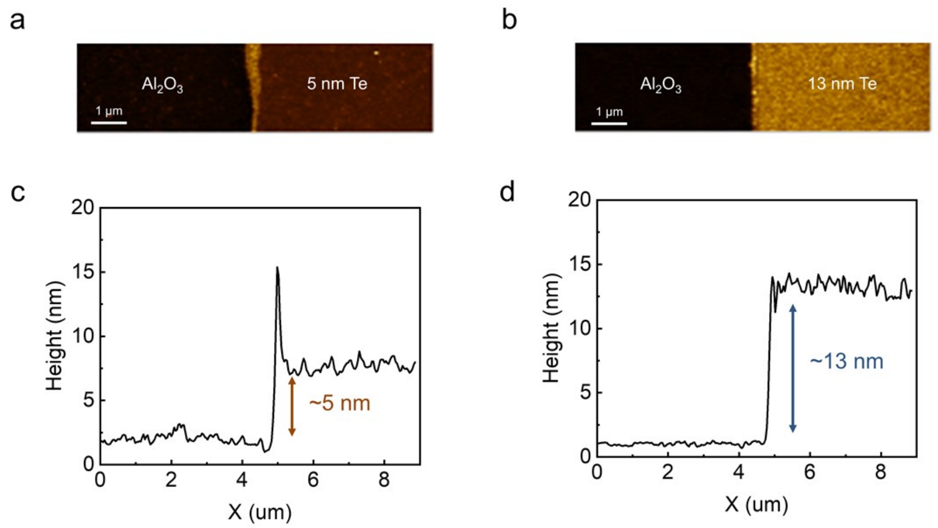

Fig. S1 Structural characterization of Te thin films via AFM. AFM topography images measured across the Al_2O_3/Te step edge for (a) 5 nm and (b) 13 nm Te films (scale bar: 1 µm). Corresponding height line profiles extracted from the AFM images, confirming step heights of (c) 5 nm and (d) 13 nm, respectively, which correspond to the actual channel thicknesses.
